# Inclusion of the Inducible Caspase 9 Suicide Gene in CAR Construct Increases Safety of CAR.CD19 T Cell Therapy in B-Cell Malignancies

**DOI:** 10.3389/fimmu.2021.755639

**Published:** 2021-10-19

**Authors:** Marika Guercio, Simona Manni, Iolanda Boffa, Simona Caruso, Stefano Di Cecca, Matilde Sinibaldi, Zeinab Abbaszadeh, Antonio Camera, Roselia Ciccone, Vinicia Assunta Polito, Francesca Ferrandino, Sofia Reddel, Maria Luigia Catanoso, Emilia Bocceri, Francesca Del Bufalo, Mattia Algeri, Biagio De Angelis, Concetta Quintarelli, Franco Locatelli

**Affiliations:** ^1^ Department of Oncology-Haematology and Cell and Gene Therapy, Bambino Gesù Children Hospital, Istituto di Ricovero e Cura a Carattere Scientifico (IRCCS), Rome, Italy; ^2^ Department of Clinical Medicine and Surgery, University of Naples Federico II, Naples, Italy; ^3^ Department of Pediatrics, Sapienza University of Rome, Rome, Italy

**Keywords:** chimeric antigen receptor (CAR T), CD19, suicide gene, immunotherapy, B-cell Acute Lymphoblastic Leukemia

## Abstract

T cells engineered with chimeric antigen receptor (CAR-T cells) are an effective treatment in patients with relapsed/refractory B-cell precursor acute lymphoblastic leukemia or B-cell non-Hodgkin lymphoma. Despite the reported exciting clinical results, the CAR-T cell approach needs efforts to improve the safety profile, limiting the occurrence of adverse events in patients given this treatment. Besides the most common side effects, such as cytokine release syndrome and CAR-T cell–related encephalopathy syndrome, another potential issue involves the inadvertent transduction of leukemia B cells with the CAR construct during the manufacturing process, thus leading to the possibility of a peculiar mechanism of antigen masking and treatment resistance. In this study, we investigated whether the inclusion of the inducible caspase 9 (iC9) suicide gene in the CAR construct design could be an effective safety switch to control malignant CAR+ B cells, ultimately counteracting this serious adverse event. iC9 is a suicide gene able to be activated through binding with an otherwise inert small biomolecule, known as AP1903. The exposure of iC9.CAR.CD19-DAUDI lymphoma and iC9.CAR.CD19-NALM-6 leukemia cells *in vitro* to 20 nM of AP1903 resulted into the prompt elimination of CAR^+^ B-leukemia/lymphoma cell lines. The results obtained in the animal model corroborate *in vitro* data, since iC9.CAR.CD19^+^ tumor cells were controlled *in vivo* by the activation of the suicide gene through administration of AP1903. Altogether, our data indicate that the inclusion of the iC9 suicide gene may result in a safe CAR-T cell product, even when manufacturing starts from biological materials characterized by heavy leukemia blast contamination.

## Introduction

Chimeric antigen receptor (CAR)-T cell therapy is one of the most innovative and revolutionary approaches in the fight against cancer, with growing evidence supporting its ability to induce complete remission of highly refractory malignancies in a significant proportion of patients (1).

It is based on autologous T cells engineered to express a CAR capable of specifically recognizing its cognate target antigen expressed on tumor cells through a single-chain variable fragment (scFv) binding domain, resulting in T-cell activation in a major histocompatibility complex (MHC)-independent manner.

To date, CAR-T cells targeting the CD19 antigen represent the first gene therapy approved for treatment of relapsed/refractory (r/r) B-cell acute lymphoblastic leukemia (B-ALL) and aggressive forms of r/r B-cell non-Hodgkin lymphoma, with up to 90% complete remission reported (1). In particular, the first two CAR-T cell products approved by the FDA (Food and Drug Administration) and EMA (European Medicinal Agency) regulatory authorities were Tisagenlecleucel (Kymriah^®^-Novartis) and Axicabtagene Ciloleucel (Yescarta^®^-Kite), but additional products are currently in an advanced stage of market authorization approval.

Despite the fact that CAR-T cell therapy represents a valid therapeutic strategy, it may induce severe toxicities, the most common being cytokine release syndrome (CRS) and CAR-T cell-related encephalopathy syndrome (CRES). Although rare, another adverse event was recently reported to potentially occur, raising the possibility of a peculiar mechanism of treatment resistance. In particular, the occurrence of an inadvertent leukemia cell transduction with CAR.CD19 vector during CAR-T cell manufacturing has been described (2,3). The CAR molecule expressed *in cis* on the surface of the leukemic cells masks the CD19 antigen, promoting the consequent expansion of a CD19**^−^** CAR^+^ leukemic clone (3). This phenomenon has been reported in a preclinical retroviral model (3), as well as in two patients treated with lentiviral-based CAR.CD19 T cells who relapsed with a CAR^+^ leukemia (2).

In this study, we investigated whether the inclusion of a suicide gene in the CAR construct could be an effective safety switch to control the event of inadvertent malignant B-cell transduction with the CAR construct, ultimately counteracting this serious adverse event.

Several safety switches have been developed, either by inclusion of transgenic enzymes selectively activated by a cytotoxic pro-drug (herpes simplex virus-thymidine kinase: HSV-TK; inducible caspase 9, iC9) or by expression of surface molecules (CD20, EGFR) that can be targeted using clinically approved monoclonal antibodies (4).

The iC9 suicide gene contains the intracellular portion of the human caspase 9 protein, fused to a drug-binding domain derived from the human FK506-binding protein. Intravenous administration of an otherwise inert small biomolecule AP1903 (Rimiducid) is able to induce dimerization of iC9, which activates the downstream apoptosome (5). In a pivotal study (6) conducted in patients given T-cell-depleted hematopoietic stem cell transplantation (HSCT) followed by post-transplant infusion of a titrated number of donor T cells transduced with iC9, it was shown that AP1903 administration can trigger chemically induced dimerization and eliminate genetically modified T cells from both peripheral blood and the central nervous system (CNS), leading to rapid resolution of graft-*versus*-host disease (GvHD) and CRS. iC9 has been used in CAR-T cell immunotherapy in several preclinical models (7), demonstrating the ability of eliminating CAR-T cells both *in vitro* and *in vivo*, although a large clinical application of this approach has been limited so far. An important aspect of iC9 is represented by its preferential killing of highly activated cells, which can be explained in view of their high expression of the transgene. As retroviral vectors preferentially integrate near transcription start sites and genes involved in proliferation (8–11), actively dividing cells typically have higher transgene expression and hence higher levels of iC9. Preclinical work suggests that transduced T cells that are not killed by AP1903 administration express an insufficient level of iC9 to allow functional activation by the dimerizing agent (5). However, it has been observed that after inducing the activation of T cells with low transgene expression that residue after the activation of iC9, it is possible to rapidly restore the AP1903 sensitivity of these cells (12).

## Materials and Methods

### Cell Cultures

The human Burkitt lymphoma cell line DAUDI (WT and iC9.CAR.CD19 DAUDI) and B-ALL cell line NALM-6 (WT and iC9.CAR.CD19) were maintained in RPMI 1640 (EuroClone, Italy) supplemented with 10% heat-inactivated fetal bovine serum (EuroClone), 2 mM of l-glutamine (GIBCO, USA), 25 IU/ml of penicillin, and 25 mg/ml of streptomycin (EuroClone), in a humidified atmosphere containing 5% CO2 at 37°C. All cell lines were authenticated by PCR single-locus-technology (Promega, USA, PowerPlex 21 PCR) analysis in “BMR Genomics s.r.l.” (Italy), and were periodically checked for mycoplasma (Venor^®^GeM Advance, MB Minerva Biolabs, UK) and surface markers expression.

### Generation and Expansion of Effector Cells

Buffy coats (BC) from healthy donors (HDs), peripheral blood (PB), and bone marrow (BM) obtained from children with B-ALL were used to isolate unfractionated mononuclear cells using Lympholyte Cell Separation Media (Cedarlane, Canada). T cells were activated with OKT3 (1 μg/ml, ThermoFisher Scientific, USA) and anti-CD28 (1 μg/ml, BD Biosciences, USA) monoclonal antibodies (mAb) with a combination of recombinant human (rh) interleukin-7 (rh-IL7, 10 ng/ml; Bio-Techne; USA) and rh-IL15 (5 ng/ml; Bio-Techne; USA). NK cells were generated from BC of HDs following a previously described method (13). After 3/4 days, T and NK cells were transduced with a γ-retroviral supernatant, in 24-well plates precoated with rhRetroNectin (Takara-Bio; Japan). T and NK lymphocytes were expanded in the presence of cytokines, in TexMacs complete medium (Miltenyi, Germany) or NK MACS medium (Miltenyi, Germany) and replenished twice a week.

### CAR Construct

T and NK cells from HD, as well as the B-cell leukemia/lymphoma cell lines DAUDI and NALM-6, were genetically modified using the retroviral construct iC9.CAR.CD19 (1e^9^ retrovirus-copies/0.5×10^6^ cells. Multiplicity of infection were quantified by Retro-X™ qRT-PCR Titration Kit from Takara, USA). The bicistronic CAR construct carries antihuman CD19-scFv from FMC63, CD8 stalk domain, CD8 transmembrane domain, 4.1bb, and CD3ζ cytoplasmic domains cloned in-frame with the iC9 suicide gene (3).

### Phenotypic Analysis

Flow-cytometry (FACS) analysis was performed to determine cell surface antigen expression; monoclonal antibodies for CD19 and CD34 (all from Becton Dickinson, NJ, USA) combined with different fluorochromes were selected according to need. FACS analysis was performed using a BD LSRFortessa X-20 cytometer (BD Biosciences, USA) and analyzed by FACSDiva software (BD Biosciences, USA). FACS sorting of CAR-transduced tumor cell lines was performed on FACSAria (BD Biosciences, USA).

### Activation of the Suicide Gene

To induce the activation of iC9 *in vitro*, cells were treated once with 20 nM of AP1903 (TOCRIS, Bio-Techne, USA), considering that this dose is in the low range of the observed Cmax for the drug (14). The percentage of residual CAR^+^ cells after exposure to AP1903 was evaluated by FACS at the indicated time points. For the *in vivo* experiments, NOD-scid IL2Rgammanull mice (NSG, The Jackson Laboratory, USA) were infused with 0.25×10^6^ iC9.CAR.CD19-DAUDI cells genetically modified with a retroviral construct to express FF-Luciferase. After tumor engraftment, documented through the IVIS imaging system, AP1903 was administered intraperitoneally from day 1 to day 28 (100 µg/day/mouse). The control cohort was infused with sterile PBS as vehicle solution. Tumors were monitored by weekly IVIS imaging analysis.

### Quantitative Real-Time PCR

The average of vector copy number (VCN) per cell was determined by real-time PCR, using a TaqMan probe designed on the retroviral construct using the Primer Express^®^ software (Applied Biosystems).

### *In Vivo* CAR^+^ Leukemia Mouse Model

Cg-Prkdcscid Il2rgtm1Wjl/SzJ (NSG) female mice were provided by Charles River and maintained in the Plaisant animal facility (Castel Romano, Rome, Italy). All procedures were performed in accordance with the Guidelines for Animal Care and Use of the National Institutes of Health (Ethical committee for animal experimentation Prot. N 088/2016-PR). At day −3, mice were infused intravenously with 0.25×10^6^ iC9.CAR.CD19-DAUDI cells genetically modified to express firefly luciferase (FF-Luc). At day 0, mice were evaluated for leukemia engraftment by the IVIS imaging system and treated with AP1903, 100 µg/day/mouse, from day 0 to day +28. Tumor growth was monitored weekly by the IVIS Imaging System, after intraperitoneal D-Luciferin (PerkinElmer, D-Luciferin potassium salt, USA) administration.

### Statistical Analysis

Unless otherwise noted, data are shown as mean ± standard deviation (SD). Student t-test (two-sided) was used to determine statistically significant differences between samples, with p-value <0.05 indicating a statistically significant difference.

Mice survival was analyzed using Kaplan-Meier survival curves, and the Log-rank (Mantel-Cox) test was used to measure statistically significant differences. No valuable samples were excluded from the analyses. Animals were excluded only in the event of death after tumor infusion, before treatment. Neither randomization nor blinding was applied. However, mice were matched in control and treatment groups based on the tumor signal. To compare the growth of tumors over time, bioluminescence signal intensity was collected in a blind fashion. Bioluminescence signal intensity was log-transformed and, then, compared using a two-sample t-test. We estimated the sample size considering no significant variation within each group of data and using a size as small as possible to obtain a significant result. The sample size was defined in order to detect a difference in averages of two standard deviations at the 0.05 level of significance, with an 80% power. Graphic representations and statistical analysis were performed using GraphPad Prism 6 (GraphPad Software, La Jolla, CA, USA).

## Results

### Suicide Gene iC9 Activation Controls Expansion of CAR+ Leukemic Cells

**Figure 1:**
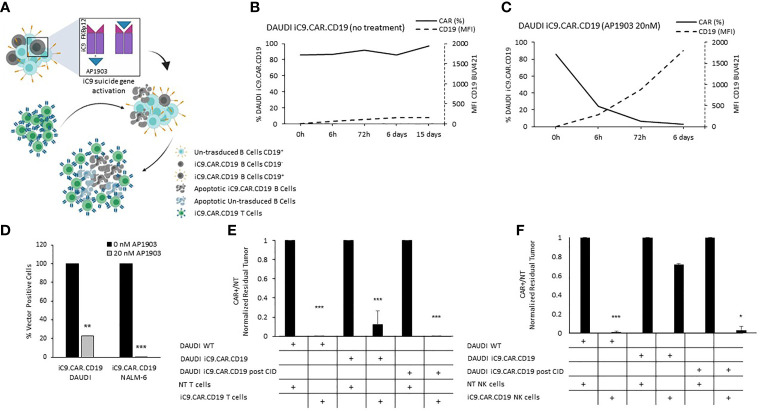
iC9 activation in iC9.CAR.CD19 lymphoma cells leads to prompt elimination of the CAR+ fraction and redetection of CD19 antigen. **(A)** Schematic representation of iC9 dimerization in iC9.CAR.CD19 lymphoma cells leading to iC9.CAR.CD19 B cells apoptosis and to redetection of CD19 antigen by iC9.CAR.CD19 T cells (created with BioRender.com). **(B, C)** iC9.CAR.CD19 DAUDI cells were treated with 0 nM **(B)** and 20 nM **(C)** of AP1903; both CAR and CD19 expressions were monitored over time by flow cytometry. **(D)** Detection of iC9.CAR.CD19 vector in tumor cells by qRT-PCR after AP1903 exposure. Reactions were performed in triplicate. Black histograms represent the positive control of reference (0 nM of AP1903), and gray histograms represent results after drug exposure (20 nM of AP1903). **(E)** A 7-day co-culture assay was carried out using un-transduced T cells or iC9.CAR.CD19 T cells and WT DAUDI cells, iC9.CAR.CD19 DAUDI cells never exposed to AP1903, and iC9.CAR.CD19 DAUDI residual after AP1903 exposure and further re-expanded (at effector/target ratio of 1:1). **(F)** A 7-day co-culture assay was carried out using un-transduced NK cells or iC9.CAR.CD19 NK cells and WT DAUDI cells, iC9.CAR.CD19 DAUDI cells never exposed to AP1903, and iC9.CAR.CD19 DAUDI residual after AP1903 exposure and further re-expanded (at effector/target ratio of 1:1) *p-value ≤ 0.05, **p-value ≤ 0.01, ***p-value ≤ 0.001.

**Figure 2:**
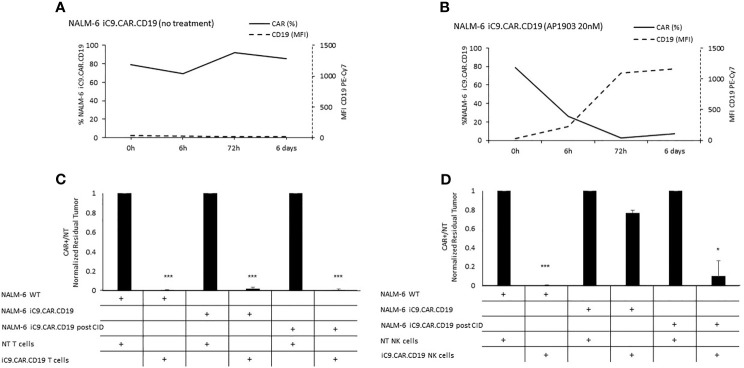
Long-term *in vitro* assays to evaluate the activity of iC9 controlling CAR.CD19-positive leukemia cell lines. **(A, B)** iC9.CAR.CD19 NALM-6 cells were treated with 0 nM **(A)** and 20 nM **(B)** of AP1903; both CAR and CD19 expressions were monitored over time by flow cytometry. **(C)** A 7-day co-culture assay was carried out between un-transduced T cells or iC9.CAR.CD19 T cells and WT NALM-6 cells, iC9.CAR.CD19 NALM-6 cells never exposed to AP1903, and iC9.CAR.CD19 NALM-6 residual after AP1903 exposure and further re-expanded (at effector/target ratio of 1:1). **(D)** A 7-day co-culture assay was carried out using un-transduced NK cells or iC9.CAR.CD19 NK cells and WT NALM-6 cells, iC9.CAR.CD19 NALM-6 cells never exposed to AP1903, and iC9.CAR.CD19 NALM-6 residual after AP1903 exposure and further re-expanded (at effector/target ratio of 1:1) *p-value ≤ 0.05, ***p-value ≤ 0.001.

**Figure 3:**
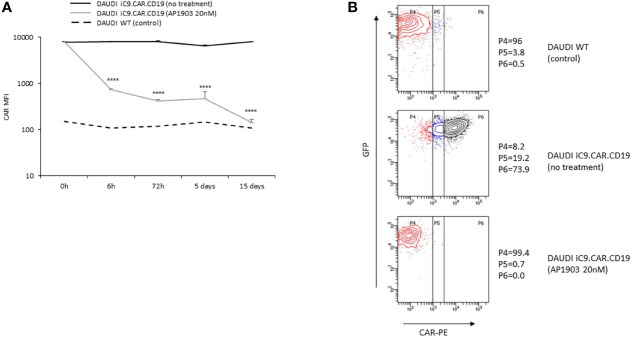
iC9 activation controls CAR+ B-cells with dim expression of transgene. **(A)** iC9.CAR.CD19 DAUDI cell line were treated with 0 nM (black line) and 20 nM of AP1903 (gray line); CAR MFI was monitored over time by flow cytometry analysis from day 0 to day 15 after treatment and compared to the control WT DAUDI cell line. **(B)** CAR expression detected by flow cytometry in the DAUDI WT cell line (negative control; top panel), iC9.CAR.CD19 DAUDI cell line (no treatment, middle panel), or iC9.CAR.CD19 DAUDI cell line treated with 20 nM of AP1903 (day +15; bottom panel) ****p-value ≤ 0.00001.

We developed a γ-retroviral vector encoding for iC9.CAR.CD19. The bicistronic construct was cloned in-frame with the iC9 suicide gene. As proof of concept, we genetically modified CD19^+^ B-leukemia/lymphoma cell lines with the bicistronic vector encoding for iC9.CAR.CD19, in order to reproduce a CAR^+^ CD19-masked leukemic clonotype (**Figure 1A**). *In vitro*, we demonstrated the prompt elimination of CAR^+^ B-leukemia/lymphoma cell lines after exposure of iC9.CAR.CD19-DAUDI and iC9.CAR.CD19-NALM-6 cells to 20 nM of AP1903. In particular, activation of the suicide gene iC9 led to a significant, early (6 h) reduction in the percentage of CAR^+^ tumor cells (**Figures 1B, C** for DAUDI cells and **Figures 2A, B** for NALM-6 cells). Prolonged, 6-day culture of AP1903-treated iC9.CAR.CD19 DAUDI cells was not associated with re-expansion of iC9.CAR^+^ lymphoma cells (**Figure 1C**). Similar results were also confirmed in the NALM-6 model: upon treatment with AP1903, no leukemia cell with high MFI of CAR expression could be detected by flow cytometry (**Figure 2B**). In particular, the CAR MFI on AP1903-treated cells (day 15) was 142 ± 22 (gray line, **Figure 3A**), a value not significantly different from un-transduced DAUDI cells (125.8 ± 20.6; dotted line), but significantly lower as compared to that of transduced cells not exposed to the dimerizing agent (7,933 ± 72, black line; p= 6E-09). Notably, AP1903 was able to eliminate not only CAR+ cells with a high CAR MFI, but also cells showing a low CAR expression (**Figure 3B** shows CAR expression before and after AP1903 treatment, respectively).

Moreover, we observed that residual leukemia cells after AP1903 exposure showed completely re-established levels of CD19 antigen expression (dotted line; **Figures 1C** and **2B**). Although qPCR analysis revealed the presence of the CAR transgene in residual blasts after AP1903 incubation, the proportion of positive cells was significantly lower as compared to that of untreated cells (**Figure 1D**, transgene positivity being observed in 22.8% of DAUDI cells, and 0.6% of NALM-6 cells).

### AP1903-Treated CAR+ B Cells Are Recognized and Eliminated by CAR.CD19 Effector Cells

Since CD19 expression was completely re-established in iC9.CAR^+^ B cells after AP1903 exposure, we evaluated whether they could be re-targeted by CAR.CD19 T cells. As shown in **Figure 1E** (DAUDI) and in **Figure 2C** (NALM-6), iC9.CAR.CD19 T cells were able to eliminate iC9.CAR^+^ tumor B cells re-expanded after AP1903 exposure. Moreover, since generating the autologous CAR-T cell product from patients with CAR^+^ B-cell relapse does not represent a feasible strategy in the clinical setting, we have also proved that *off-the-shelf*, third-party, HD-derived iC9.CAR.CD19 NK cells (13) are able to significantly control iC9.CAR.CD19^+^ B cells rescued after AP1903 treatment (**Figure 1F** for the DAUDI model, and **Figure 2D** for the NALM-6 model).

### *In Vivo* Suicide Gene Strategy to Control CAR+ Leukemia

**Figure 4:**
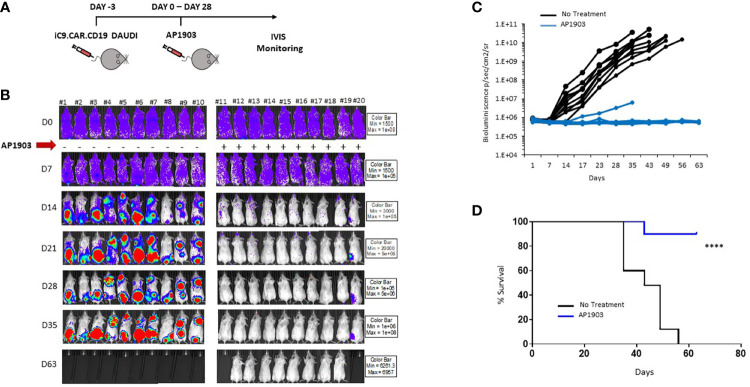
iC9 activation is able to control *in vivo* expansion of CAR-positive lymphoma in a xenograft mouse model. **(A)** Schematic representation of the experimental design, with iC9.CAR.CD19-positive/FF-Luciferase-positive DAUDI cells infused at day −3. At day 0, mice were evaluated for leukemia engraftment and treated with 100 µg/mouse of AP1903 from day 0 to day 28. **(B)** Bioluminescence images of each control untreated mouse and each AP1903-treated mouse. Mice were monitored for more than 30 days after AP1903 withdrawal. **(C)** Bioluminescence values over time for each treated mouse in the two cohorts, untreated (black lines) or AP1903-treated (blue line) mice. **(D)** Kaplan-Meier survival curve analysis of leukemia-bearing mice untreated (black line) or AP1903-treated (blue line). ****p-value ≤ 0.00001. The only mouse (#20) showing a persisting positive signal at IVIS analysis after AP1903 administration was sacrificed at day+43 together with a negative control (#11), to characterize the leukemia cells.

We proved *in vivo* the ability of the suicide gene iC9 to eliminate CAR+ leukemia expansion using AP1903. NSG mice were infused with iC9.CAR.CD19 DAUDI cells at day −3 (**Figure 4A**); after tumor engraftment, the dimerizing drug AP1903 was intraperitoneally administrated from day 0 to day 28 (**Figure 4A**). iC9 activation resulted in complete eradication of CAR+ leukemia in 9 out of 10 studied mice (**Figures 4B, C**). Moreover, AP1903 administration resulted in survival of all mice without leukemia but one, even after stopping drug administration, with 9 out of 10 mice showing absence of leukemia cell progression until day 63 (endpoint of the experiment; **Figure 4D**).

## Discussion

In this study, we provide evidence that the inclusion of the iC9 safety switch in a CAR vector represents a powerful and effective rescue strategy in case of inadvertent CAR.CD19^+^ blast generation. In comparison to other suicide genes, the iC9 safety switch is non-immunogenic and is characterized by a rapid mechanism of action once activated by the binding with AP1903 (15). Our data confirm previously published *in vitro* (16) and *in vivo* studies (17) showing that AP1903 is highly efficient in eliminating the great majority of cells highly expressing iC9, while sparing cells with a lower level of iC9 expression. Indeed, in the context of genetically modified T cells, patients who received an infusion of haploidentical, donor-derived iC9-T cells after HSCT and were subsequently treated with Rimiducid to control GvHD, showed 1% of residual iC9-T cells a few hours after AP1903 administration. The residual iC9-T cells were characterized by a remarkably dim expression of the transgene, possibly related to a low level of T-cell activation. Elimination of iC9-T cells could be enhanced by T-cell activation during repeated AP1903 administrations (17). By contrast, when iC9 is expressed in tumor cells, AP1903 is able to induce massive and rapid apoptosis, leading to a significant elimination of tumor cells (16). With our CAR system, in case of an unlucky inadvertent transduction of leukemia blasts with iC9.CAR.CD19, we demonstrated that CAR+ B cells can be efficiently eliminated by exposure to AP1903 and that the expression level of the CAR in rescued leukemic cells after treatment is negligible. Leukemia cells that could not be eliminated by AP1903 and lacking a high expression of CAR on their surface express a standard level of CD19 antigen on their surface, this results in the possibility of efficiently targeting B-cell leukemia/lymphoma elements through CAR.CD19 T cells and CAR.CD19 allogenic NK cells.

The results we obtained in our animal model corroborated *in vitro* data, since iC9.CAR.CD19^+^ DAUDI cells could be controlled *in vivo* by the activation of the suicide gene through administration of AP1903. In particular, NSG mice infused with iC9.CAR.CD19 DAUDI cells were treated with intraperitoneal administration of AP1903 from day 0 to day 28. iC9 activation resulted in complete eradication of CAR^+^ B cells in the great majority of the mice (90%), without leukemia relapse after withdrawal of the drug for a prolonged period of time.

Altogether, our data support the hypothesis that for patient-derived CAR-T cell products, the inclusion of the iC9 suicide gene in the construct represents an important safety strategy, able to control and eliminate leukemia cells in case of inadvertent blast-cell transduction during CAR-T cell manufacturing, and meets the medical need of increasing the safety profile of CAR-T cell gene therapy. Moreover, the iC9 suicide gene could contribute to successfully treat other CAR-T cell therapy associated toxicities, such as CRS and CRES, if not adequately controlled by conventional pharmacological treatment. In this respect, we are now conducting a clinical trial in which we treat patients with B-ALL using CAR-T cells transduced with a retroviral construct including iC9 (NCT03373071).

## Data Availability Statement

The original contributions presented in the study are included in the article/supplementary material. Further inquiries can be directed to the corresponding author.

## Ethics Statement

The animal study was reviewed and approved by the Ethical Committee of the Italian Ministry of Health, Prot. N 088/2016-PR.

## Author Contributions

FL, BDA, and CQ are the co-last author of the paper. SM, MG, and IB are the first authors of the paper. CQ, BDA, and FL designed experimental studies, supervised the project conduction, analyzed the data, and wrote the manuscript. IB, MG, SM, SDC, MS, SC, ZA, AC, RC, VP, FF, and SR developed the *in vitro* models and performed the *in vitro* experiments. IB, BDA, and CQ performed the *in vivo* experiments. CQ, IB, and BDA cloned the retroviral vector. IB, MG, SM, and MS performed FACS analysis. FDB, MA, MLC, EB, and FL provided healthy donor material, medical advice, and expertise in childhood B-ALL and B-NHL. All authors contributed to writing the paper and approved the final version of the manuscript.

## Funding

The experimental work was supported by grants awarded by GR-2016-02364546 (BA), RF-2016-02364388 (FL), Accelerator Award–Cancer Research UK/AIRC–INCAR project (FL), Associazione Italiana Ricerca per la Ricerca sul Cancro (AIRC)-Special Project 5×1000 no. 9962 (FL), AIRC IG 2018 id. 21724 (FL), MFAG 21979 (CQ), Ricerca Corrente (CQ, BA), Ministero dell’Università e della Ricerca (Grant PRIN 2017 to FL); Italian Healthy Ministry project on CAR T RCR-2019-23669115 (Coordinator FL), Independent Research grant AIFA (FL: 2016 call).

## Conflict of Interest

The authors declare that the research was conducted in the absence of any commercial or financial relationships that could be construed as a potential conflict of interest.

## Publisher’s Note

All claims expressed in this article are solely those of the authors and do not necessarily represent those of their affiliated organizations, or those of the publisher, the editors and the reviewers. Any product that may be evaluated in this article, or claim that may be made by its manufacturer, is not guaranteed or endorsed by the publisher.
